# Role of Mirikizumab in the Treatment of Inflammatory Bowel Disease—From Bench to Bedside

**DOI:** 10.3390/jcm14031001

**Published:** 2025-02-05

**Authors:** Michael Colwill, Samantha Baillie, Jennifer Clough, Richard Pollok, Andrew Poullis, Kamal Patel, Sailish Honap

**Affiliations:** 1 Department of Gastroenterology, St George’s University Hospital NHS Foundation Trust, London SW17 0QT, UK; michael.colwill@nhs.net (M.C.); s.baillie1@nhs.net (S.B.); jennifer.clough@nhs.net (J.C.); richard.pollok@nhs.net (R.P.); andrewpoullis@nhs.net (A.P.); kamal.patel@stgeorges.nhs.uk (K.P.); 2 Institute of Infection and Immunity, City St George’s, University of London, London SW17 0RE, UK; 3 School of Immunology and Microbial Sciences, King’s College London, London WC2R 2LS, UK

**Keywords:** mirikizumab, inflammatory bowel disease, IL-23p19 inhibitor

## Abstract

Mirikizumab is a monoclonal antibody directed against the p19 subunit of interleukin (IL)-23 to inhibit its interaction with the IL-23 receptor. IL-23 is a key cytokine involved in initiating and perpetuating the inflammatory cascade in inflammatory bowel disease (IBD). Mirikizumab is the first agent from the novel anti-IL-23p19 drug class to be licensed for ulcerative colitis and the first to present long-term endoscopic, histologic, symptomatic, and quality-of-life outcomes. More recently, the VIVID trial programme has led to the approval of mirikizumab in moderate to severe Crohn’s disease. This review explores the history of its development, discusses key immunopharmacological properties unique to the drug, and details the available clinical trials and real-world evidence supporting its use in IBD.

## 1. Introduction

The last decade has seen a significant increase in the number of advanced therapy (AT) options available to clinicians for the treatment of inflammatory bowel disease (IBD). Historically, pharmacological treatment options were limited to 5-aminosalicylic acids, thiopurines, and corticosteroids, but that changed in the 1990s with the emergence of the first clinical trials demonstrating the efficacy of infliximab, a monoclonal antibody targeting tumour necrosis factor-alpha (anti-TNF⍺) in both Crohn’s disease (CD) [1] and later ulcerative colitis (UC) [2]. Since these landmark trials, the therapeutic armamentarium has grown considerably to include other anti-cytokine drugs, such as Janus kinase inhibitors and interleukin (IL)-12 and/or IL-23 inhibitors, and anti-leucocyte trafficking agents that include the anti-integrins and sphingosine-1-phosphate receptor modulators. However, there remains a significant proportion of patients who fail to respond to, lose response to, or are intolerant of these ATs, creating an unmet need for novel therapies.

IL-23 has been identified as a dominant regulatory cytokine involved in both innate and adaptive immune systems and as playing a critical role in multiple immune-mediated inflammatory diseases (IMIDs) [3,4]. It has been implicated in the pathogenesis of psoriatic skin and joint inflammation, rheumatoid arthritis, and IBD [5]. Since its discovery, targeting IL-23 has been an area of significant research interest when developing new treatments for IMIDs, and ustekinumab, a monoclonal antibody that inhibits both IL-12 and IL-23, has been shown to be effective in several IMIDs, including IBD [6,7,8,9].

IL-23 is a heterodimer of the p40 and p19 subunits. Whilst p40 is also found in IL-12, p19 is unique to IL-23, and this specificity makes it an attractive target for novel therapies. This has led to the development of several agents that act as p19 inhibitors (p19i) and the accumulating safety and efficacy clinical trial data are promising. Mirikizumab (Omvoh, Lilly) was the first p19i to be licenced for the treatment of UC following the LUCENT trial programme [10] and is being appraised by regulators for use in CD in Europe and the United Kingdom after receiving approval from the FDA. This review examines the pharmacology of mirikizumab and the history of its drug development, the clinical trial and real-world data underpinning its use in IBD, and a discussion on where it will likely be positioned in treatment algorithms for IBD.

## 2. Understanding the Pharmacological Basis of Mirikizumab

IL-23 is a member of the IL-6 cytokine family [11], and it was discovered in the late 1990s, with subsequent studies identifying IL-23 as a major effector cytokine involved in innate and adaptive immune responses. It is a driver of aberrant inflammation in autoimmune disorders of the skin [12], joints, lungs, and gut. The p40 subunit is shared with IL-12, with the p19 subunit unique to IL-23 [13]. IL-23 is produced by immune cells, including activated dendritic cells, in response to toll-like receptor (TLR) signaling [11]. Myeloid cells that express Fc-gamma receptor 1 (FcγRI), or CD64, have been identified as a primary source of IL-23 in inflamed gut tissue. Genetic variants in the region of IL-23 and IL-23R have been associated with altered susceptibility to the development of IBD, and high serum IL-23 levels have been identified in patients, particularly in subjects with co-existent arthritis [14,15]. Together, this suggests a role for the IL-23 pathway as an attractive therapeutic target in IBD.

IL-23 plays a key role in the differentiation and maintenance of CD4+ T helper 17 (Th17) cells, a T cell subset identified as critical to the pathogenesis of both UC and CD [13,16,17]. IL-23 induces strong proliferation of Th17 cells, resulting in the production of proinflammatory cytokines, including IL-17A, IL-17F, IL-21, and IL-22 [13]. Furthermore, IL-23 can act on neutrophils to induce the production of pro-inflammatory IL-17 and IL-22 [18]. Despite success in treating immune-mediated skin conditions [19], blockade of IL-17 has been shown either to be ineffective or to worsen CD in clinical trials [20,21], suggesting an additional protective role for IL-17 in supporting gut barrier function [22]. Serum IL-23 levels correlate with disease activity in UC and a reduced ratio of tolerogenic regulatory CD4^+^ T cells to proinflammatory Th17 cells in peripheral blood [16]. Blockade of IL-23 holds the potential, therefore, to modulate the function of Th17 cells in autoimmune disease whilst permitting immune responses to invasive pathogens [23].

The IL-23 receptor is composed of the IL-23R and IL-12Rβ1 subunits. Whilst IL-23R is responsible for initiating downstream intracellular signalling pathways, IL-12Rβ1 stabilises binding through interactions with the p40 subunit of IL-23 [24]. Binding of IL-23 leads to recruitment and activation of intracellular Janus (JAK2) and tyrosine kinases (TYK2), followed by phosphorylation and nuclear translocation of signal transducer and activation of transcription (STAT) 3 and 4 transcription factors [11].

### 2.1. The Development of Clone 9F2.25.38: Mirikizumab

Given the importance of IL-23 in the inflammatory cascade, multiple attempts at discovering new therapeutic agents targeting this pathway have been undertaken. The development of what became mirikizumab started with in vitro models using BALB/c mice that were immunised with human IL-23. Spleen cells from these mice were then harvested and sorted using fluorescence activation to identify those with IL-23 binding activity. These were then cultured for 2 weeks with EL4-B cells following which they were assayed for positive binding to IL-23 and a lack of binding to IL-12. Reverse-transcription polymerase chain reactions were subsequently used to isolate heavy and light chain variable region genes from the desired cells, which were subsequently cloned into murine antibody expression vectors. The resulting antibodies were then assessed and characterised for antigen binding affinity and their ability to block IL-23R binding using either human or murine spleen cell assays. This process identified clone 9F2 which completely inhibited IL-23 binding to its receptor without binding to IL-12. This clone was then successfully humanised and optimised for affinity and biophysical properties, such as chemical stability, resulting in four high-affinity variants. Further in vitro studies assessing protein characteristics such as affinity, aggregation, and expression levels resulted in clone 9F2.25.38 being designated as mirikizumab [23].

Further evaluation using in vitro animal studies demonstrated that mirikizumab binds to IL-23 with high specificity, with no measurable binding to IL-12, preventing interaction between IL-23 and IL-23R but not the interaction with IL-12Rβ1 [23,25]. Human T cell assays confirmed that mirikizumab was able to block IL-23-induced IL-17 production whilst preserving the function of IL-12. These results were replicated in studies on mice and cynomolgus monkeys, demonstrating good efficacy and safety and allowing the progression to human clinical trials.

### 2.2. Pharmacokinetics of Mirikizumab

**Figure 1:**
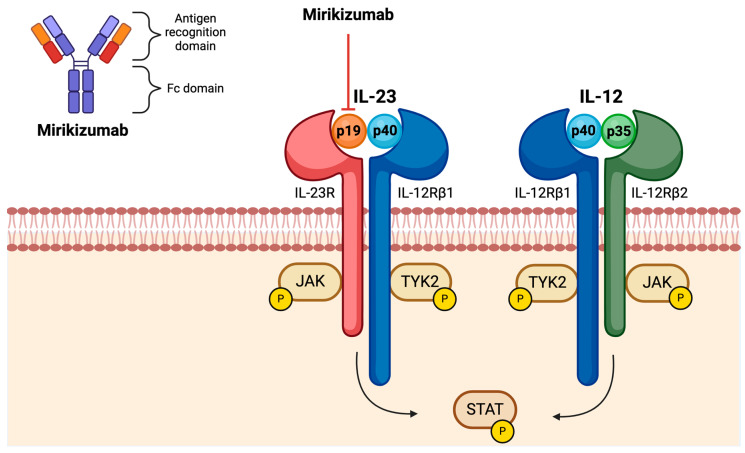
Mirikizumab is a humanised IgG monoclonal antibody specific to the p19 subunit of IL-23. Interleukin (IL)-23 is a heterodimeric cytokine comprised of p19 and p40 subunits. The p40 subunit is also shared by the heterodimeric cytokine IL-12, which is additionally comprised of a p35 subunit. The receptor for IL-23 is composed of an IL-12Rβ1 and IL-23R chain. The receptor for IL-12 is composed of two different subunits, IL-12Rβ1 and IL-12Rβ2. Binding of IL-23 or IL-12 to their respective receptor results in conformational changes in the receptor, which induce autophosphorylation of Janus kinase (JAK) 2 and tyrosine kinase (TYK) 2, leading to activation of signal transducers and activators of transcription (STATs).

Mirikizumab (Omvoh, Lilly, Indianapolis, United States, LY3074828) is a neutralising humanised IgG4 monoclonal Ab directed against the p19 subunit of IL-23 (Figure 1) and was the first p19i approved in the United Kingdom for moderate-to-severe UC in 2023. Mirikizumab induction is administered as three four-weekly 300 mg intravenous infusions, followed by four-weekly maintenance dosing of 200 mg delivered subcutaneously from week 12. Mirikizumab, unlike the other p19i, also offers an extended 24-week induction or re-induction if patients lose response. The drug has a half-life of 9.3 days, shorter than that of risankizumab (21–29 days) and guselkumab (17 days [25]), with time to peak mirikizumab concentration being five days for subcutaneous dosing. Although a quarter of patients in phase III trials developed anti-mirikizumab antibodies (ADAs) during treatment, immunogenicity did not seem to affect drug availability, with only 2.6% of patients having reduced serum mirikizumab concentrations in the presence of ADAs [26]. Previous studies have demonstrated that low serum albumin levels in the context of active IBD can affect monoclonal antibody trafficking and clearance [27,28]. Whilst lower serum albumin concentrations have been associated with higher mirikizumab clearance, the effect size was small and is unlikely to have a clinically meaningful effect on mirikizumab levels [29]. Studies have also suggested dose adjustment for weight is not required [26].

## 3. Pivotal Clinical Trials

### 3.1. Ulcerative Colitis

**Figure 2:**
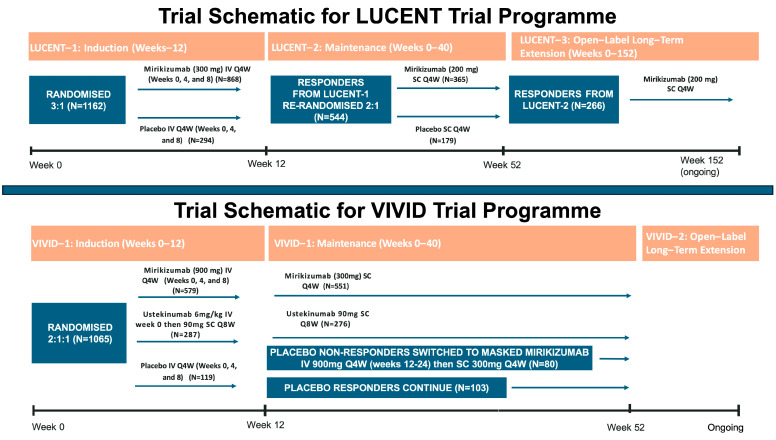
Schematic of the trial designs for LUCENT and VIVID trial programmes.

LUCENT-1 was a phase-3 induction randomised control trial (RCT), which investigated 1281 patients with moderate-to-severely active UC and randomised them in a 3:1 ratio to receive intravenous mirikizumab (300 mg) or placebo (no active treatment) every 4 weeks for 12 weeks (Figure 2) [10]. Individuals had failed glucocorticoids, immunomodulators, or AT and were excluded if prior therapy included an IL-23 inhibitor (ustekinumab, tildrakizumab, guselkumab, or risankizumab). At week 12, mirikizumab was superior at achieving the primary endpoint of clinical remission (24.2% vs. 13.3%; 11.1% difference; 99.875% CI 3.2 to 19.1; *p* < 0.001) and all major secondary endpoints, including urgency numerical rating score (NRS) (*p* < 0.001). A single induction trial was needed to demonstrate efficacy across all primary and secondary endpoints with 99.875% confidence intervals by using a familywise error of 0.00125 (instead of the standard hypothesis testing threshold of *p <* 0.05).

In the LUCENT-2 maintenance trial [10], 544 responders from LUCENT-1 were re-randomised 2:1 to receive subcutaneous mirikizumab (200 mg) or placebo (no active treatment) in a 2:1 ratio from weeks 12 to 52. Mirikizumab non-responders (*n* = 272) received three doses of intravenous open-label mirikizumab (300 mg) every 4 weeks and were reassessed for clinical response 12 weeks later. Those who had responded received open-label subcutaneous mirikizumab (200 mg) every 4 weeks through week 40. Responders to the placebo in the induction period continued to receive a blinded placebo in the maintenance period. Any patient with a loss of response after week 12 of the maintenance period discontinued maintenance mirikizumab or placebo and received three doses of open-label mirikizumab (300 mg), 4 weeks apart, as rescue therapy. At week 40, mirikizumab was superior at maintaining clinical remission compared to placebo (49.9% vs. 25.1%, 23.2% difference; 95% CI, 15.2 to 31.2; *p* < 0.001) with 97.8% of mirikizumab-treated patients in remission being glucocorticoid-free. Among the 272 patients reinduced with mirikizumab after inadequate primary response, 53.7% had a clinical response, and 11.4% had clinical remission by week 12 (week 24 overall). Clinical remission was maintained in 72.2% of these patients, and 36.1% had clinical remission at week 40. Secondary clinical, endoscopic, and histological endpoints also favoured mirikizumab over placebo, with a significantly greater number of individuals meeting the secondary endpoints. Improvement from baseline in urgency NRS remained stable throughout the maintenance trial in the mirikizumab group, whereas patients re-randomised to placebo lost some of the improvement gained during the induction trial.

LUCENT 3 is an ongoing single-arm, open-label, long-term extension study assessing the safety and efficacy of mirikizumab [30]. Individuals from LUCENT 1 or 2 who would benefit from further treatment received subcutaneous mirikizumab (200 mg) every 4 weeks, with interim analysis results published after a continuous treatment period of 104 and 152 weeks [30]. Those treated with a placebo were not included in the analyses. Approximately 25% of patients in the intention-to-treat population had missing data at week 152, either sporadically missing or due to early discontinuation; therefore, endpoint analysis is provided for non-responder imputation (NRI—patients who discontinued treatment or were missing endpoint assessments were treated as non-responders), observed cases (patients with missing data were not included and missing data were not imputed) and modified non-responder imputation (m-NRI—discontinuation treated as non-response but sporadic missing data imputed). Using NRI, OC, and m-NRI, week 52 responders were in response at week 152 in 71.6%, 94.9%, and 81.6% of cases and in remission in 49.5%, 56.1%, and 65.5% of cases, respectively. Data were similar for biologic-failed and biologic-naive subgroups.

The mirikizumab-responder group had a ≥3 change in urgency score and ≥1 change for both stool frequency and rectal bleeding that was maintained through week 152. Over 80% of individuals had ≥30% improvement in abdominal pain from baseline to week 152.

During the first 52 weeks, 23.6% of mirikizumab-treated patients had anti-drug antibodies; however, less than 2% of mirikizumab-treated patients had antibody titre ≥1:160 associated with lower trough mirikizumab concentrations (<0.511 μg/mL, 5th percentile) and reduced clinical response. During the LUCENT-3 extension, only a further 0.6% developed antibodies.

Safety signals and adverse event (AE) rates were low throughout the LUCENT trial programme and are discussed in greater detail below.

These studies notably included participants up to the age of 80, who are an often-excluded demographic in clinical trials. Given the rising prevalence in the older population, this is particularly meaningful as the observed safety profile can be generalised to the older population.

### 3.2. Crohn’s Disease

The phase 3 randomised, double-blind, placebo and active-controlled VIVID-1 trial compared mirikizumab to placebo (no active treatment) and ustekinumab in 1065 individuals with moderately to severely active Crohn’s disease. Participants were randomised 6:3:2 to mirikizumab (900 mg IV at weeks 0–12, then 300 mg SC every 4 weeks at 12–52 weeks), ustekinumab (~6 mg/kg IV dose, then 90 mg SC every 8 weeks to week 52) or placebo (Figure 2) [31]. Note that the dose of mirikizumab for induction was three times higher for CD than for UC, based on the dose-ranging phase II trials.

Both co-primary endpoints of the study were met: patient-reported outcome (PRO) clinical response at week 12 plus week 52 endoscopic response (mirikizumab 38.0% vs. placebo 9.0%, *p* < 0.000001) and week 12 PRO clinical response plus week 52 Crohn’s Disease Activity Index (CDAI) clinical remission (mirikizumab 45.4% vs. placebo 19.6%, *p* < 0.000001). Mirikizumab demonstrated superiority over placebo across all secondary endpoints, and significant reductions in abdominal pain, stool frequency, C-reactive protein (CRP), and faecal calprotectin were observed as early as weeks 4–6.

The trial also showed non-inferiority to ustekinumab in CDAI clinical remission at week 52 after accounting for multiplicity (*p* = 0.113), although superiority in endoscopic response was not achieved (*p* = 0.51) despite higher numerical response rates in the mirikizumab arm. Statistically significant reductions in CRP and faecal calprotectin were observed with mirikizumab compared to ustekinumab. Additionally, mirikizumab demonstrated numerically higher, but not statistically significant, rates of clinical remission by PRO and corticosteroid-free CDAI remission at week 52 compared to ustekinumab. Numerical, but not statistical, superiority was also demonstrated for mirikizumab over ustekinumab for CDAI and endoscopic response rates in those with previous biologic failure (Table 1).

The safety profile of mirikizumab was favourable, with lower rates of adverse events (AEs) compared to placebo. Anti-drug antibodies were detected in 12.6% of patients treated with mirikizumab, predominantly low-titre and transient, with a neutralizing activity that did not significantly affect drug efficacy.

## 4. Real-World Experience with Mirikizumab

Given the recent licensing of mirikizumab in UC, little real-world clinical data are available for its use outside of a clinical trial setting. A small study in 17 UC patients reported a modest reduction in clinical activity and faecal calprotectin, with a reduction in median Simple Clinical Colitis Activity Index (SCCAI) from 7 to 5 at weeks 8–12 of treatment [32]. Notably, 16 patients had received at least one previous biologic therapy, 12 had received ≥2, and four patients received ≥4, suggesting that mirikizumab may be effective in a biologic-experienced real-world cohort. No adverse events were reported in the short follow-up period.

## 5. Safety

A key strength of ustekinumab, the first IL-12 and IL-23 antagonist approved to treat IBD, is its robust long-term safety profile, which has been demonstrated in both clinical trials [6,7] and from real-world evidence [33]. Given their similar mechanism of action, there has been an expectation that p19i will have similar safety profiles, allowing widespread use without high levels of treatment cessation or complications. Thus far, the safety data from the randomised control trials that brought mirikizumab to the market have found low levels of serious adverse events (SAE) in both UC and CD, offering a positive outlook for its long-term use.

### 5.1. Ulcerative Colitis

For UC, the LUCENT trial programme found low rates of SAEs in both the induction and maintenance phases (2.1% and 3.3%, respectively, when excluding worsening UC), which were lower than those treated with placebo [10]. Common adverse events included nasopharyngitis, arthralgia, headache, and rash and were present at levels comparable to other advanced therapies (Table 2).

The LUCENT 1 and 2 trials found that serious infection, described as an adverse event (AE) of interest, was similar or lower in those treated with mirikizumab compared to placebo. With regards to opportunistic infections in the mirikizumab-treated cohorts, six patients developed herpes zoster infection, which was unrelated to corticosteroid use; four developed candidiasis; four developed cytomegalovirus, and one was diagnosed with intestinal tuberculosis. Only one patient treated with a placebo developed an opportunistic infection in the form of a herpes zoster infection. The rates of malignancy were low with four reported cancers in the 1217 mirikizumab-treated patients compared to none in the placebo-treated group; two were diagnosed with colorectal adenocarcinoma, one with gastric cancer and one with non-melanomatous skin cancer. One potential signal was with regards to elevations in liver enzymes which were more frequent in mirikizumab treated patients compared to placebo. In LUCENT 1 and 2, 27 patients developed deranged liver enzymes compared to nine in the placebo cohort, although none of these patients met the criteria for Hy’s law. Depression was reported in four patients during the maintenance phase who received mirikizumab and in no patients who received a placebo.

Safety data from the open-label extension LUCENT 3 study included data from 285 patients at week 152 [30]. Common AE rates were similar to the LUCENT 1 and LUCENT 2 data and 8.8% of patients developed an SAE, with 5.3% of patients discontinuing treatment due to an AE. The commonest was the development of COVID-19 infection (22.4%), reflective of the fact it was held during the pandemic and the worsening of UC (15.9%). Rates of infusion or injection-site reactions were higher with mirikizumab than placebo: 4 (0.4%) patients in the induction trial and 34 (8.7) in the maintenance trial. Regarding SAEs of interest, there was one reported malignancy, six opportunistic infections, five cerebrocardiovascular events, and one major adverse cardiac event. Eleven patients treated with mirikizumab developed elevations of alanine aminotransferase, but none met the criteria for Hy’s law. Three patients reported depression, and one patient attempted suicide, although it should be noted they had a history of suicide attempts prior to enrolment in the trial. There was one death from thrombotic thrombocytopenic purpura on day 463 of the trial.

### 5.2. Crohn’s Disease

The VIVID-1 study compared mirikizumab to both placebo and ustekinumab in treating moderate to severe CD and found that the rates of AEs, SAEs, and discontinuation were similar in mirikizumab and ustekinumab populations and both were lower than the placebo group [31]. The most common AE were COVID-19 infection, anaemia, arthralgia, headache, upper respiratory tract infection, nasopharyngitis, and diarrhoea, and all occurred with a higher adjusted incidence ratio in the placebo group. Infusion site reactions (0.2%) and injection site reactions (10.8%) were higher in mirikizumab than in the placebo (0 and 6.5%, respectively, for the placebo cohort). Seven patients treated with mirikizumab developed opportunistic infections: one oral candidiasis, one typhoid fever, and five herpes zoster infections compared to none in the placebo cohort. Mild elevations in liver enzymes were seen in 6.2% of mirikizumab-treated patients, but none met the criteria for Hy’s law. Three participant deaths occurred: one pulmonary embolism in the placebo cohort, one sepsis in the ustekinumab arm, and one worsening of CD in a placebo non-responder who switched to mirikizumab at week 12. No deaths were considered to be drug-related.

### 5.3. Pregnancy and Lactation

There are very limited data available with regard to the safety of mirikizumab in pregnancy and lactation. Pre-clinical studies performed on 30 pregnant cynomolgus monkeys found no adverse development events to those born of mothers given mirikizumab at 79 times the maximum human dose during organogenesis whilst pregnant. There were no mirikizumab-related adverse events in mothers, foetuses, or infants followed up with 6 months after birth, and the overall incidence of embryonic/foetal loss was within the historical control data. Whilst mirikizumab was detected in all infants at 28 days after birth, the concentration in maternal milk was not assessed. There was also no evidence of an impact on fertility in monkeys administered 30 times the maximum human dose.

In the previously described clinical trials assessing mirikizumab in humans, 28 pregnancies were reported. There were three spontaneous abortions, and six elective terminations occurred. Eight infants were born without major congenital abnormalities, with one born preterm at 34 weeks [34]. The outcomes of four pregnancies were unknown, and seven were still in utero at the time of data analysis [34].

There are no data on maternal use of mirikizumab and transmission in breast milk or its impact on the infant. Given its large molecular size, it is unlikely to be excreted in large amounts in breast milk [35]; however, it is known that human immunoglobulins are excreted in breast milk early after birth, and therefore, it is possible that mirikizumab is passed into the breast milk post-partum [36]. The impact of this on the infant is unknown.

Whilst data are limited, its safety profile is expected to be similar to that of other monoclonal antibodies in IBD, and decisions should be made on a case-by-case basis after honest and clear discussions with the patient.

### 5.4. Safety from Real-World Evidence

The relatively short time that mirikizumab has been licenced means that there is only a small amount of real-world safety data currently available. A study presented by Lande et al. [32] at ECCO 2024 followed 17 patients with UC over a four-month period who were treated with mirikizumab and found no AE reported during this period.

In summary, with regards to safety, whilst larger real-world studies over longer time periods are still required in order to fully understand the long-term safety profile of mirikizumab, the evidence thus far available demonstrates low overall risks to patients of SAE and a comparable safety profile to ustekinumab.

## 6. Positioning

Targeting IL-23 and IL-12 holds an established position in the paradigm of IBD treatment through the widespread use of ustekinumab, which has been proven to be an efficacious and safe therapy. Given this, the question of when to use mirikizumab, particularly in preference to ustekinumab, remains unanswered.

The LUCENT trials previously discussed have demonstrated that mirikizumab is an effective therapy for UC, including in patients who have previously been treated with an anti-TNF⍺ or JAK inhibitor. Sub-group analysis from these data demonstrated that in patients with one anti-TNF⍺ failure, significantly more patients achieved clinical response at week 12 compared to placebo (64.4% vs. 34.1% *p* = 0.001), clinical remission at week 52 (44.3% vs. 17.2%, *p* = 0.017), and symptomatic remission at week 52 (63.9% vs. 34.5%, *p* = 0.005) [37]. When comparing this to a sub-group analysis of patients with previous biologic failure (32.6% of whom had failed both an anti-TNF⍺ and vedolizumab) from the UNIFI trial, which assessed ustekinumab in UC, at week 8, 57.2% of those treated with ustekinumab achieved clinical response (vs. 27.% placebo, *p* = 0.001), and at week 44, 39.6% were in clinical remission (vs. 17% placebo, *p* = 0.001) [38]. Whilst not a direct head-to-head comparison, these data potentially suggest a slightly greater efficacy of mirikizumab in biologic-exposed patients.

Given the lack of direct comparative data, network meta-analyses (NMA) have attempted to inform on the relative superiority of AT, although to date, few have included mirikizumab. One study, performed under a Bayesian framework by Dignass et al. [39] in October 2024, compared the randomised clinical trial data from multiple currently licenced advanced therapies for UC. They compared clinical response, remission, and mucosal healing in both induction and maintenance phases and, importantly, given the known impact of a previous anti-TNFα on the efficacy of advanced therapies, sub-divided patients into biologic/JAKI naïve and biologic/JAKi exposed cohorts. In the naïve cohort, upadacitinib and infliximab were superior compared to all other therapies for clinical response and remission during induction but in the maintenance assessment, mirikizumab was found to be superior compared to anti-TNFα, vedolizumab, tofacitinib, ozanimod and upadacitinib 15 mg and comparable to ustekinumab and upadacitinib 30 mg. In those patients with previous biologic/JAKi treatment, in both induction and maintenance, upadacitinib (45 mg and 30 mg, respectively) was superior for clinical response and remission. Mirikizumab was broadly comparable to ustekinumab across both the naïve and exposed cohorts. There was no significant difference in this study when assessing SAE. An important negative point was that it did not include risankizumab in the analysis as it was not at the time licenced for UC. Ananthakrishnan et al. [40] have since produced an NMA, which included risankizumab, and found that, whilst mirikizumab and ustekinumab were similarly effective at inducing remission in UC in biologic-naïve patients, both were inferior to risankizumab regardless of previous biologic exposure. However, further NMAs or head-to-head trials are required to corroborate these findings.

With regards to CD, the VIVID-1 study suggested that when comparing clinical and endoscopic endpoints, there was a numerical trend towards a greater response with mirikizumab than ustekinumab. The study also demonstrated non-inferiority to ustekinumab when assessing clinical remission by CDAI. An NMA by Vuyyuru et al. [41], which included data from 20 clinical trials focusing on endoscopic outcomes, found that in CD, anti-TNFα agents appear to be superior, followed by JAKi and p19i, and in biologic-exposed patients, both JAKi and p19i appear to be the most effective therapies. There is a paucity of data with regard to mirikizumab in CD, and further data from RCTs, head-to-head studies, and NMAs are required before we can clearly define the positioning of mirikizumab in CD.

The lack of currently available real-world evidence also means that when deciding which treatment to use, we are reliant on NMAs, even with the known limitations, particularly with regard to the heterogeneity of study design, duration, and endpoints. Taken overall, the available data suggest that mirikizumab is an effective treatment in UC; however, it may be inferior to risankizumab, which was approved for use in UC by the Food and Drug Administration in June 2024 and the National Institute of Health and Care Excellence in the UK in August 2024, and head-to-head studies are needed.

As well as the comparative data, there are other factors that will be relevant when positioning mirikizumab such as cost and practicalities of administration. The newly licenced ustekinumab biosimilars offer significant cost savings to healthcare providers, and it may, therefore, be mandated by funding bodies that these agents are used ahead of mirikizumab. The requirement for intravenous loading adds further cost and will put additional strain on infusion suite capacity; therefore, agents that can be loaded subcutaneously, such as the guselkumab [42], may be preferred by some healthcare systems.

## 7. Conclusions

Mirikizumab has been shown to be efficacious for UC and CD, and it promises to be an important AT in the coming years. The reassuring safety data suggest it may be a suitable treatment for elderly patients and those with multiple comorbidities. Future areas of research include ascertaining treatment positioning in the landscape of IBD therapies and understanding its safety and efficacy in special populations, such as extreme age classes, different ethnicities, and those with multiple comorbidities. Other research areas include its potential use in advanced combination therapy regimens and identifying appropriate treatment response predictors.

## Figures and Tables

**Table 1:** Breakdown of primary endpoints in the pivotal trials comparing results for biologic naïve patients and those with previous biologic failure. In the VIVID data, biologic failure was either an anti-TNF antibody or an anti-integrin antibody. For the LUCENT data, biologic failure also included those with previous treatment failure to tofacitinib.

	Biologic Failed			Biologic Naive		
	Placebo	Mirikizumab	Ustekinumab	Placebo	Mirikizumab	Ustekinumab
	*n* (%)	*n* (%)	*n* (%)	*n* (%)	*n* (%)	*n* (%)
VIVID-1: N	97	281	139	102	298	148
Clinical Response by PRO week 12 and Clinical Remission by CDAI Week 52	12 (12.4)	122 (43.4)	-	27 (26.5)	141 (47.3)	-
Clinical Response by PRO week 12 and Endoscopic Response by SES-CD Week 52	6 (6.2)	103 (36.7)	-	12 (11.8)	117 (39.3)	-
Clinical Remission by CDAI Week 52	12 (12.4)	144 (51.2)	58 (41.7)	27 (26.5)	169 (56.7)	81 (54.7)
Endoscopic Response by SES-CD Week 52	6 (6.2)	126 (44.8)	55 (39.6)	12 (11.8)	154 (51.7)	78 (52.7)
LUCENT 1: N	118	361	-	171	492	-
Clinical Remission Week 12	10 (8.5)	55 (15.2)	-	27 (15.8)	152 (30.9)	-
LUCENT 2: N	64	128	-	114	229	-
Clinical Remission Week 40	10 (15.6)	59 (46.1)	-	35 (30.7)	118 (51.5)	-

PRO: Patient-reported outcome. CDAI: Crohn’s Disease Activity Index. SES-CD: Simple Endoscopic Score for Crohn’s Disease.

**Table 2:** Key safety data from the LUCENT trial programme [10] and VIVID trial [31].

	LUCENT Trials—UC				VIVID Trial—CD		
	Induction Trial		Maintenance Trial				
	Placebo (*n* = 321)	Mirikizumab (*n* = 958)	Placebo (*n* = 192)	Mirikizumab (*n* = 389)	Placebo (*n* = 211)	Mirikizumab (*n* = 630)	Ustekinumab (*n* = 309)
Any adverse event	148 (46.1)	426 (44.5)	132 (68.8)	251 (64.5)	154 (73.0)	495 (78.6)	239 (77.3)
Any adverse event, excluding ulcerative colitis	141 (43.9)	421 (43.9)	116 (60.4)	241 (62.0)	N/A		
Serious adverse event	17 (5.3)	27 (2.8)	15 (7.8)	13 (3.3)	36 (17.1)	65 (10.3)	33 (10.7)
Serious adverse event, excluding ulcerative colitis	7 (2.2)	20 (2.1)	10 (5.2)	13 (3.3)	N/A		
Discontinuation rate due to adverse event	23 (7.2)	15 (1.6)	16 (8.3)	6 (1.5)	20 (9.5)	32 (5.1)	8 (2.6)
Death ^†^	0	0	1 (0.5)	0	1 (0.5)	0	1 (0.3)
Malignancy ^‖^	0	2 (0.2)	1 (0.5)	1 (0.3)	1 (0.5)	2 (0.6)	0
Non-melanoma skin cancer (not included in malignancy count)	0	0	1 (0.5)	0	1 (0.5)	1 (0.2)	0

^†^ There were two deaths during the follow-up period for the LUCENT induction trial: one from sudden cardiac arrest and one from disseminated intravascular coagulation. The death in the placebo group during the maintenance trial was due to coronavirus disease 2019. ^‖^ In the mirikizumab group during the LUCENT induction trial, both cancers were colon adenocarcinoma. During the maintenance trial, nonmelanoma skin cancer (basal-cell carcinoma) occurred in one patient in the placebo group and gastric cancer in one patient in the mirikizumab group.

## Abbreviations

Anti-TNFα	Anti-Tumour Necrosis Factor Alpha
AE	Adverse events
AT	Advanced Therapy
CD	Crohn’s disease
FDA	Food and drug administration
IBD	Inflammatory bowel disease
JAKi	Janus Kinase inhibitors
MACE	Major adverse cardiovascular event
P19i	IL23 p19 Inhibitors
RCT	Randomised controlled trial
SAE	Serious adverse events
UC	Ulcerative colitis
